# Patients’ experiences in the use of mobile health clinics in KwaMachi rural area of KwaZulu-Natal, South Africa

**DOI:** 10.1016/j.dialog.2023.100164

**Published:** 2023-12-31

**Authors:** Ms Zama Portia Nkosi

**Affiliations:** Faculty of Social Sciences, School of Sociology and Population Studies, Walter Sisulu University, Mthatha, Eastern Cape, South Africa

**Keywords:** Mobile health clinics, Rural healthcare access, Patient experiences, Qualitative research, KwaZulu Natal, South Africa

## Abstract

**Background:**

A study was conducted to evaluate the role of mobile health clinics (MHCs) in providing healthcare services in rural areas, focusing on the population of KwaMachi in KwaZulu Natal, South Africa. The objective of the study was to explore the level of health promotion and health-seeking behaviour in KwaMachi, understand the services provided by the MHCs, and assess the experiences of individuals accessing healthcare through these clinics.

**Methods:**

The study used qualitative research methods, including face-to-face interviews and focused group discussions. A sample of 20 patients, with equal representation of males and females across different age groups, was selected using purposive and convenience sampling methods. Two themes were developed: positive experiences and negative experiences. The collected data was analyzed using manual thematic analysis.

**Findings:**

The findings of the study revealed that MHCs serve as the main entry point to the national healthcare system in rural areas, but they offer limited services, which contributes to the ongoing struggle of rural communities to access primary healthcare. While respondents generally had positive experiences with MHCs, some negative aspects need to be addressed. These included concerns about privacy during consultations, the unavailability of essential medications, limited services offered, lack of doctors, and inadequate management of clinical records leading to diagnostic inaccuracies.

**Conclusion:**

The study highlights the challenges faced by rural communities in accessing healthcare services and the role of MHCs in addressing these challenges Based on these findings, the study concludes that there is a need for sustainable MHC programs that address the specific needs and preferences of the local population.

**Recommendations:**

These insights are of value to policymakers seeking to enhance the impact of MHCs in improving healthcare access and outcomes in rural areas. By looking at areas of improvement in the services provided by MHCs, including addressing privacy concerns, ensuring the availability of essential medications, and improving the management of clinical records. The study provides valuable insights for policymakers to enhance the effectiveness of MHC programs in rural areas.

## 1 Background to the study

Health care is vital to human existence. Without good health, people are unable to enjoy other aspects of life such as recreational activities, financial stability and stable employment [1]. A recent report by the United Nations International Labour Organization (ILO) reveals that a significant number of people living in rural areas lack access to vital healthcare services, highlighting a stark disparity compared to those living in urban areas [2]. Rural areas in South Africa refer to the geographic regions characterized by low population density and limited urbanization [3]. These are typically marked by a predominantly agrarian economy, with agriculture being the primary source of livelihood for the local population. Rural areas are often situated far away from major cities and urban centres and may lack some of the infrastructure and services commonly found in urban areas such as paved roads, public transportation and extensive healthcare facilities [2,4]. According to the 2001 population census, the proportion of rural dwellers in South Africa (SA) was 42.5 percent with Kwazulu Natal (KZN) having the largest proportion at 54.0 percent [3]. Unfortunately, people residing in rural areas are consistently confronted with significant barriers when attempting to access primary health care services. By access, the researcher refers to the ability for individuals or communities to obtain and utilize essential medical care and treatment when needed [5]. It encompasses various dimensions, including proximity to healthcare facilities, affordability of the services, the acceptability of care in cultural and social terms.

The study was conducted at a mobile health clinic in the KwaMachi area, located in Harding which falls under the UMziwabantu Municipality in the Ugu District in KwaZulu Natal South Africa. KwaMachi is a chiefdom with a unique history. The area was for the most part neglected as it was not clear whether it constituted part of the province of KwaZulu-Natal or Mpondoland. While it has been identified as part of KwaZulu-Natal, this has not had much impact on the way that people in this district are perceived and the delivery of basic services. This is evident in the provision of basic services to the community and in the lack of infrastructural development in the area. In this rural area, there is still a lack of necessary amenities, such as proper housing, running water and poor sanitation amongst other things. One of the major reasons for the delay in service delivery is the harsh topography that makes the district inaccessible.

Access to primary health care service is a fundamental human right that should be available to all individuals, regardless of their geographic locations. Access to healthcare and equitable distribution of health services are not just desirable but essential for achieving good health within populations and this is true for South Africa [6]. The apartheid policies in South Africa resulted in underdeveloped, fragmented and underserviced rural areas, negatively impacting the health of the population [7,8]. To address this issue, the government recognised the need for constitutional reform to ensure equal rights for all citizens, including access to healthcare services [7]. By guaranteeing human dignity, equality, and freedom through the constitution’s Bill of Rights Charter, the government aimed to unify the fragmented healthcare system and allocate resources effectively [9]. This commitment to equitable healthcare was further reinforced by the Reconstruction and Development Programme, which prioritises healthcare initiatives [10]. As part of this programme, MHCs were introduced. The introduction of mobile healthcare vehicles by the South African government was a crucial step towards ensuring accessible healthcare for all. Mobile health clinics (MHCs) offer adaptable and feasible alternatives for delivering healthcare services to marginalised and at-risk populations, including those who are geographically isolated [2,11].

In this study, the concept of mobile health clinics refers to the “mobile single cab vans” used by the National Department of Health to deliver healthcare services in rural areas in KwaZulu Natal (KZN). While it is not precisely known when the first mobile medical facility started functioning in these KZN communities, there has been a progressive increase in the number and demand for MHCs in the country [12]. Across the country, people living in rural areas suffer limited access and other socio-economic barriers to health care and mobile medical facilities are changing that by making health care accessible to communities [13]. Mobile vans emerged as a critical solution to the long-standing problem of inadequate and often non-functional primary healthcare facilities in rural areas [12]. They have seamlessly integrated into the province's healthcare system, addressing the pressing issue of limited access to healthcare services that have long plagued rural communities. The observed limited access to healthcare services can be attributed to the enduring obstacles inherent in poverty, thereby placing the rural communities at a significant disadvantage relative to their urban counterparts [14,15].

In addition to the aforementioned concern, it is imperative to acknowledge that rural areas particularly in KZN are confronted with a substantial prevalence of chronic ailments, thereby compounding the existing healthcare predicament [16,17]. The persistent prevalence of chronic diseases, encompassing, cancer, diabetes, tuberculosis and HIV/AIDS, persist as a formidable challenge within these communities [16]. A study conducted by McIntyre et al. (2018) emphasizes the gravity of this issue, shedding light on the significant mortality burden imposed by chronic disease within rural areas in KZN [18].

It is on this awareness that this study sought to evaluate the role of the MHCs also known as the medical vans in providing quality healthcare services in rural areas, specifically focusing on the health needs of the population of KwaMachi, a rural area in KwaZulu Natal, South Africa. The study aims to explore the level of health promotion and health-seeking behaviour in KwaMachi, understand the services provided by the medical vans, and assess the experiences of individuals accessing healthcare through the MHCs. The study also aims to determine the perceived service quality and patient satisfaction with the mobile vans, as well as how these adjust to meet the various healthcare needs of rural communities.

## 2 Literature review

Mobile health clinics are important as they offer significant healthcare services where access to care is inadequate [19]. In a recent study in Malawi, it was found that women and children are disproportionately affected by health inequalities. [20]. To address the issue, non-governmental organizations have been working alongside with government to provide healthcare services to underserved populations in remote areas. The aforementioned non-governmental organizations are responsible for the inception of MHCs aimed at providing complementary healthcare services to children with ailments in these areas [20]. The MHCs can offer regular services and cover significant distances in central Malawi, however, the operation of these MHCs is impeded by challenges such as inadequate infrastructure and adverse weather conditions [20].

In another study undertaken by Neke et al. (2018), it was found that mobile health clinics have positively impacted the coverage of essential maternal and child health services. This suggests that MHCs have a role in improving healthcare access in certain areas. Despite this increased coverage the mobile vans continue to face financial, human resources and logistics constraints. These constraints hinder their ability to provide healthcare services effectively. Furthermore, MHCs have been proven to be adaptable, with some European countries employing them for non-communicable disease management, while they serve as a critical point of access to the public primary healthcare system [22].

To increase uptake at the MHCs, it is crucial to raise awareness within the community about the services provided [23]. Additionally, addressing issues of cost for some clients is essential to ensure a steady uptake. This can be achieved by improving the availability of essential services and efficient community care workers [19]. It is worth noting that immunization, antenatal care and child development monitoring have been successfully carried out in most MHCs [24,25]. However, the COVID-19 pandemic worsened the situation, leading to MHCs not being deployed during the national emergency [26].

In conclusion, authors [11,21,27,28] agree that mobile vans are beneficial for rural populations, but equitable health provision remains a concern when compared to urban areas. Despite being resource-intensive, mobile vans are necessary for remote areas. However, as noted in the Malawi case study, the sustainability of the MHC model is context-dependent and uncertain [20]. To ensure effective delivery of health services, proper planning and resource mobilization should accompany MHCs. Continuous monitoring and evaluation of cost and effectiveness are essential [26]. Additionally, in some countries, there is a lack of tools to measure, improve and communicate the impact of MHCs.

### 2.1 Theoretical framework: Health Equity Framework

To address the issue of the lack of tools to measure, improve and communicate the impact of MHCs, and draw a foundation for this study, I draw upon the Health Equity Framework (HEF). This theory is widely recognised in the field of public health and it highlights the importance of achieving equitable access to healthcare services for all, irrespective of socioeconomic factors, geographical location or other determinants of health [29].

**Fig. 1:**
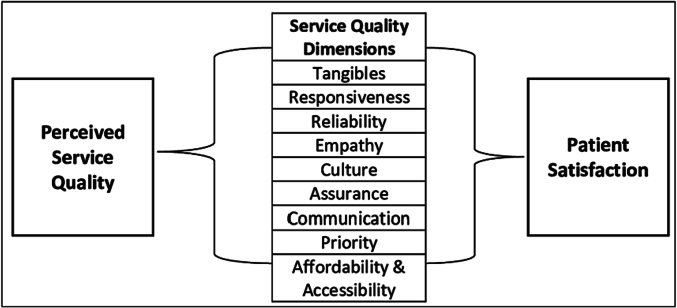
Health equity model. Source: Ahenkan and Aduo-Adjei (2017) [30].

The HEF is adopted to guide the exploration of the study on how MHCs contribute to reducing the disparities in health care access and outcomes amongst vulnerable populations in rural areas particularly, in KZN South Africa. It also informs my examination of the various dimensions of access which include accessibility, acceptability and affordability, to better understand the complexities of health care delivery through MHCs in the context of health equity. By employing the HEF, the study recognises and validates the agency of patience in shaping their interactions with the MHCs, allowing the study to delve into the intricacies of their experiences, choices and decision-making processes [29]. Fig. 1 below is an illustration of how HEF provides a valuable lens through which to understand patient experiences when accessing the MHCs by focusing on the principles of service quality dimensions such as acceptability, affordability and accessibility to understand patient satisfaction.

The HEF offers a comprehensive and patient-centered approach to understanding patients' experiences with MHCs. It encourages the exploration of broader social, economic and cultural dimensions that influence well-being [31].

## 3 Methods

The research study adopted the interpretivism paradigm and followed the qualitative study design. This choice is driven by the inherent complexity of understanding lived experiences, perspectives and meanings attributed to healthcare equity in the context of MHCs [32]. With healthcare equity, the researcher aimed to gauge the principles such as accessibility, acceptability, affordability, availability and utilization of the MHCs [33].

**Fig. 2:**
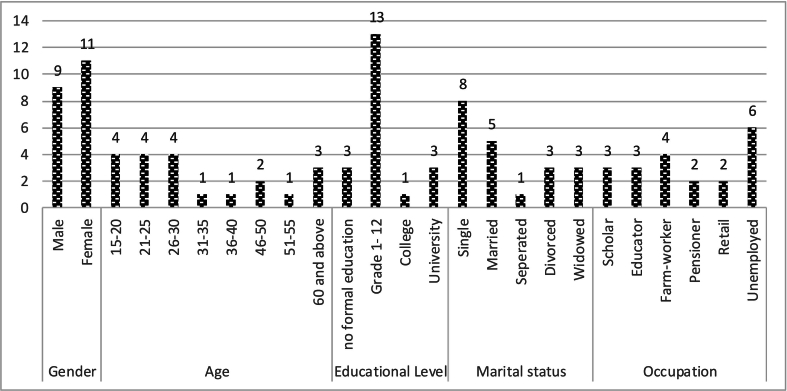
Demographic characteristics of participants.

Primary data was collected by the lead researcher, who had the experience with undertaking a research through face-to-face interviews and focused group discussions. The sample selection was done using purposive and convenience sampling methods. The study included a sample of 20 patients, with equal representation of males (n=10) and females (n=10) across different age groups, of which three [3] had young children under the age of ten. As depicted in Fig. 2 below, the research sample consisted of participants between the ages of 18 and 69, with the majority being youth. Participants for the focus group discussions were selected from participants of the in-depth interviews to shed more light on the perspectives and experiences of patients. Of the 20 participants who were part of the in-depth interviews, fourteen [14] had the opportunity to be part of the focus group discussions. The majority of the respondents were 18 years and above, had average levels of education and were unemployed. In addition, most were currently not married.

A triangulation method through the use of both face-to-face interviews and focus groups was used to minimize any form of bias that distorts the results [34,35]. The data was made more reliable by using both semi-structured in-depth face-to-face interviews and focus groups with the users of the KwaMachi rural area. Trustworthiness and authenticity were reinforced through overted observations. Participants were made aware of the presence of the researcher. The interviews were audio-recorded and transcribed. Manual thematic analysis was used to analyse the collected data. The full written thesis was disseminated to the participants giving them the opportunity to review their transcripts^1^.

### 3.1 Ethical considerations

The study was approved by the Higher Degree’s Ethics Committee at the University of KwaZulu Natal (UKZN). The UKZN research ethics guidelines were adopted to ensure no exposure to risk and confidentiality. The researcher observed all ethical protocols to ensure the preservation and the well-being of the research participants. Before conducting the fieldwork, the researcher made preliminary visits to KwaMachi. During the first visit, a meeting was held with the chief in charge to seek permission to conduct the study. This involved locating MHC sites and selecting research participants. The informed consent form were signed by participants to ensure that their participation was voluntary and that they had a right to discontinue the interviews when they felt discomfort [36]. A short demographic questionnaire was administered to ascertain respondents’ age, gender, marital status, and occupation. During the interviews, the beneficiaries were asked about their medical history and how using the mobile health clinics has helped them improve their health status. Data were analysed using the manual thematic analysis technique.

## 4 Results

The results of the patient experiences in the use of MHCs are discussed below. The study found that MHCs have been operational in this community for the past ten years and are used by community members of different ages and genders. Furthermore, the participants visited the mobile health clinic to treat different ailments. The ailments included but were not limited to contraception (including condoms), regular checkups for diabetes, and checking of results for TB screening. Their experiences were classified into two categories, either positive or negative based on the elicited response. In the positive category, there were two themes identified, firstly, what was realized is that the mobile health clinic plays an important role in linking people to the national public health care system and secondly, MHCs provide solely needed healthcare services. On the negative category, the negative category, five themes were identified, firstly, there was a lack of privacy during consultation, secondly, lack of doctors, thirdly, inadequate operating hours and infrastructure and lastly, limited service offered. The themes are discussed in detail in the paragraphs that follow.

### 4.1 Positive experiences at the mobile health clinic

#### 4.1.1 An entry point to the public health care system

The respondents stated that they use MHC because of different reasons. Firstly, they stated that MHCs help everyone in need of access to health care services in their community, although not all the required services are always available. For instance, eye care and dental services were not available at the clinic. For those patients who want to do the tooth extraction, they are referred to the hospital. Secondly, unlike the hospital where there is an administrative fee; access to mobile health clinics is free. Thirdly, they argued that mobile health clinics are convenient as it saves them time and money. They do not have to travel long distances to a fixed clinic. For them travelling long-distance includes walking in the bushy forests and this is not safe as it exposes them to the possibility of being mugged and raped.

#### 4.1.2 Provision of sorely needed services

There was a general feeling that people utilized MHCs because when they go there for consultations, they are given the attention they require, although the medicine they require is not always available. The services offered range from childhood vaccinations; family planning; diabetes; health check-ups; HIV testing; and tuberculosis testing. However, ailments like asthma and epilepsy patients would only be treated there and then when there has been an episode, but no further referral is done to that patient and no further treatment is given to the patient that will assist in curbing asthma or epilepsy attacks when at home. It is only when the patient has been hospitalized that they are prescribed monthly chronic medication.

Respondents who are mothers expressed satisfaction with the child vaccination services offered by the MHCs. Asked to describe a typical day at the MHC one respondent said that they arrived early so that they could leave early. Overall, the experiences seemed to be quite positive as outlined in the following comment:

*“We wake up in the morning and prepare to come to the mobile health clinic (umahamba nedlwana). One has to come very early to be the first in the queue*. *I arrive around 08h30 in the morning and then I wait for 10h00 which is the time of arrival of the mobile health clinic at the Zamani crèche. I know when I come early, I will be finished before 11h00 and then I go home to do other household chores (P1).*

One patient described their experience as positive as it was uplifting because she felt that it saved her time.

*Before we start with the consultations, the nurse will make us start by singing and then we all pray. This is done as a routine every morning before receiving our health care. Then the nurse who is doing administration asks for our cards. One nurse will do the consultation and the other will distribute medication which makes the process quicker. This saves us time. We get to finish early and go home to do other chores” (P2).*

Travelling to the nearest hospital can be costly especially if one is unemployed let alone the elderly who survive on their pension money that supports a number of their extended family members including grandchildren. People in rural areas are often faced with the challenge of a lack of resources, particularly money. Difficult choices have to be made between using money to visit a healthcare facility and using it to buy food or necessities for the household. Many households in this community survive on the pension grants as a result how the money is spent within the household becomes a critical factor. Respondents highlighted that the mobile clinic brings health care to their doorsteps.

### 4.2 Negative experiences at the mobile health clinics

In general, respondents were happy with the mobile health clinics but still highlighted some negative experiences. These centred on privacy during the consultation, non-availability of essential drugs, limited services offered, and a lack of doctors and follow-ups on previous diagnoses.

#### 4.2.1 Lack of privacy during consultation

Privacy during the consultation was amongst the most serious concerns that people encounter at mobile clinics. This was the main concern of male respondents. They stated that this prevented them from reporting all their health concerns to the nurse. One respondent noted that other people can hear what they are talking about with the nurse. They fear that people will spread the news of their state of health in the community. As a result, they are reluctant to share an accurate and complete history of their diagnosis because of the fear of being judged and consequently do not get all the medical help they need. There is a need for the MHC structure to be planned in a manner that ensures that it meets the emerging needs of community health. Furthermore, the KwaMachi Community is still a traditional society where certain customs are observed, and certain diagnoses are still treated as taboo. There is a lot of secrecy around health issues in the community. Hence, the respondents expressed the need for MHC sites to provide optimal audio and visual privacy.

On the other hand, they felt that MHCs are not the ideal facility for receiving some health care services such as a pap smear, pregnancy testing and HIV testing, amongst others because the van is too small to accommodate such services. One respondent said that it was embarrassing when a urine sample is required since there is no secluded place and toilets are often located far from the van.

#### 4.2.2 Lack of required medication

The issue of shortage of required medication at the MHC was also raised by respondents. One female respondent said:

“*The nurses do give out medication if they have enough on that day but the issue of lack of medication is happening more regularly now than before*” (P6).

Interruption in drug supplies is likely to result in a negative perception of healthcare services.

Another respondent added that if there were doctors at the MHCs, the situation would be different.

#### 4.2.3 Lack of doctors at MHCs

The lack of qualified medical doctors is a real challenge in addressing the full range of health problems. Respondents feel that doctors should also serve at mobile health clinics. The shortage of qualified doctors in rural areas impacts the quality of health care. One respondent noted:

*“I went to a mobile health clinic for a checkup because I had a very severe eye problem but they could not help me as I am speaking to you my eye is still sore. I am going to ask them to write me a doctor’s letter to take to the hospital”* (PI)*.*

On the one hand, the lack of doctors, compromised the quality of health care that the patients received because doctors are seen to provide higher quality health care than nurses. Unfortunately, the shortage of doctors is dominant even in fixed hospitals and clinics and the MHCs are no exception.

On the other hand, the nurses at the MHC rarely provide referral letters for patients to be seen by doctors at the local hospitals and when the letter is written for referral; often it does not detail the clinical condition at the time of referral. Hospitals rely on referral letters when deciding which case to schedule as a priority for their hospitals. As a result, provision for health care is delayed and by the time the patient gets the letter to go and see a doctor their health has deteriorated.

#### 4.2.4 Lack of awareness of services offered as a barrier to access

There is a need for public awareness of the services rendered on that particular MHC. The respondents mentioned that when they are at the fixed health clinic they may come for consultation on one health ailment, but they leave with information and knowledge on how to curb other sicknesses because of the posters and pamphlets that are distributed at the clinic. This was a recurring theme, particularly among the young patients. The community recommended that the pamphlets should be made available at the MHC.

#### 4.2.5 Inadequate operating hours and infrastructure

There was general dissatisfaction with the operating hours. The majority of the respondents felt that a once-a-week visit by the mobile health clinic was not sufficient as sickness is unpredictable. They argue that the mobile health clinic should be visited at least twice or thrice a week. Asked how many hours the mobile health clinic spends at their site; they said it depends on the number of people that are there at the time. It serves the people who are there, even if it is three people, and then moves on to the next site. Furthermore, it is difficult for mobile health clinics to operate in bad weather conditions. The clinic is meant to visit once a week, but the community will go even for weeks without treatment if the weather is bad and sometimes people have to hold on to their pain until the mobile health clinic comes. While the respondents in KwaMachi felt that the MHC site should be moved to a weather-friendly site, the challenge is that there is no suitable venue since they do not have a community hall.

## 5 Discussion

The discussion revolves around the role of Mobile Health Clinics (MHCs) in bringing healthcare closer to people, particularly in rural areas of South Africa. The study highlights that for a long time travelling long distances and the cost associated with travelling has hindered people’s access to health centres, emphasizing the importance of MHCs in addressing this issue. In another study done in Mpumalanga, it was found that costs associated with accessing healthcare sometimes amounted to up to 60 per cent of the household income particularly for families particularly for families with disabled members [15].

Consistent with the literature, is that MHCs play a significant role in bringing health care closer to the people [27]. Thus, the study used people’s experiences to assess the role played by MHCs in bringing health care closer to the people. This is important as morbidity is high amongst infants and children in rural areas [37]. The study revealed that women of childbearing age are frequent users of the MHC. The study also revealed that fewer males than female patients use the MHC. This is mainly because the majority of males in this district place more value on traditional medicine than Western medicine. While the youth used the MHC, they said that they are sometimes afraid to ask the nurses questions relating to issues of reproductive and sexual health. They feel that the MHC should provide pamphlets written in a local language explaining these issues.

The context in which people live cannot be ignored as it has a huge impact on them remaining healthy after they have received health care at the MHCs. The context in which people live is crucial for their overall health, supporting the idea that MHCs should focus on preventative screening rather than just treatment. This supports the argument by Carmack et al. that MHCs are largely providing preventative screening more than treatment of the diagnosed ailment [38]. It also mentions the need for tailored services that respond to the specific needs of the population.

Coordination of services and restructuring of the patient sitting positions within these medical vans are suggested to improve privacy and efficiency. Haskins et al., suggested that in such cases it is important that the services are well coordinated [39]. In this case, it is a matter of restructuring and arranging the sitting positions of the patients so that there is audio privacy. Where possible big medical trucks can be provided instead of single-cab medical vans.

The study also reveals that women of childbearing age are frequent users of MHCs, while fewer males utilize these services due to a preference for traditional medicine. This supports the argument by Carmack et al. that services offered in MHCs must not be limited but are tailor-made for that specific population, but they must be impactful and respond to the dynamically evolving community needs [38]. The youth express a need for more information on reproductive and sexual health. The discussion concludes by acknowledging the ongoing challenges in the healthcare system, including the burden of communicable and non-communicable diseases.

In another study conducted in the Eastern Cape, it was discovered that when there is bad weather the mobile clinics do not come [39,40]. While much has been done to improve the healthcare system in South Africa, the growing burden of communicable and non-communicable diseases continues to undermine the achievement of healthcare services [41,42]. The study revealed that there is a shortage of medical doctors in rural areas as such some conditions remain untreated for a long time before the patient is transferred to the hospital. Mahboube et al., proposes that if this situation is to be addressed there should be less co-dependency on doctors and some clinical tasks be given to nurses and all relevant clinical staff [43].

### 5.1 Limitations of the study

This research study had some limitations. Firstly, the nature of the methodology used; the results cannot be generalized to other populations. However, the results have the potential to inform policy and practice. Another limitation of the study was the sensitivity of the research focus: people’s experience at MHCs. Some respondents felt uncomfortable speaking about their vulnerable situations and their health status. Finally, due to the exploratory nature of the study, it would have been preferable to include a larger sample; however financial constraints rendered this impossible.

## 6 Conclusion and recommendations

The research revealed a consensus that people in KwaMachi greatly appreciate the convenience offered by MHCs. This appreciation is mainly because for many in this community, MHCs represent the primary and sometimes the only option for receiving primary healthcare services. One implication that emerges from the findings is the need for a strategic redesign of the MHCs. Specifically, there is a need to consider larger vans that can provide greater privacy during patient consultation and thus allowing the patients talk freely about their illnesses to be treated. Furthermore, this will also allow space for the van to carry enough medication and provide additional health care services. This aligns with the broader national healthcare goals in South Africa, particularly the 2030 mandate, which seeks to ensure equal access to care for all citizens. In conclusion, the evaluation of patient experiences is, therefore, an important component of health services improvement. This will ensure that the government achieve its 2030 mandate of providing equal access to care for all.

## Authors contributions

ZN collected the data and conducted data analysis and the write-up.

## Declarations

None

## Consent for publication

The author consented to the publication of the manuscript after reading and approving the final version of the manuscript.

## Funding

I confirm that we did not receive any funding for this research study.

## Declaration of competing interest

The authors declare the following financial interests/personal relationships which may be considered as potential competing interests:

Ms Zama Portia Nkosi report financial support and article publishing charges were provided by Walter Sisulu University. Ms Zama Portia Nkosi reports a relationship with Walter Sisulu University that includes: employment.

## Data Availability

The data that has been generated and subsequently analyzed in the course of this study are regrettably not accessible to the public, as the participants involved have provided their informed consent solely for the specific purposes outlined within the confines of this study.

## Footnotes

1 This method, as defined by Pelto, entails the utilization of multiple research methodologies as a means to evaluate and ascertain the veracity and dependability of data-gathering techniques within the domains of the social and behavioral sciences [34,35]. This study used different data collection methods including face to face interviews and focus groups.
